# The Mediation Effects of Social Media Usage and Sharing Fake News about Companies

**DOI:** 10.3390/bs12100372

**Published:** 2022-09-30

**Authors:** Daniel-Rareș Obadă, Dan-Cristian Dabija

**Affiliations:** 1 Department of Communication Sciences and Public Relations, Alexandru Ioan Cuza University of Iași, 700506 Iași, Romania; 2 Department of Marketing, Faculty of Economics & Business Administration, Babeș-Bolyai University Cluj-Napoca, 400084 Cluj-Napoca, Romania

**Keywords:** concentration, online trust, perceived control, sharing fake news, social media usage, time distortion, social networking sites (SNS)

## Abstract

Trust in social media information is gaining in importance and relevance for both companies and individuals as nowadays contemporary society is confronted with a wave of fake news about daily life situations, brands, organizations, etc. As it becomes more difficult to accurately assess social media information and to determine its origin or source, as well as to be able to double-check information spread across different Social Networking Sites (SNS), businesses must understand how individuals’ perceived control, concentration, and time distortion enhances the social media usage, thus allowing them to correctly assess online information. Therefore, the scope of the paper is to assess, based on a conceptual model, the antecedents of trust in online information about companies by considering users’ perceived control, concentration, and time distortion, while browsing social media networks and sharing fake news about companies in SNS. With the help of an online survey, data was collected from social media users, later being analysed with SmartPLS. The findings suggest that social media usage and sharing of fake news mediate the relationship between users’ perceived control, concentration, and time distortion (i.e., flow characteristics) and trust in online information about companies.

## 1. Introduction

As companies relied even more over the past year on social media for promoting their own brands, products, and corporate image, SNS developed as new points of contact with customers and other key stakeholders such as suppliers, retailers, government, NGOs, etc. Worldwide there are approximately 4.62 billion active social media users that spent more than 12 trillion hours on social networking sites [1]. Considering that social media sites have rapidly grown in popularity, now they are widely considered as a critical strategic component of an organization’s competitiveness and survival [2,3]. Relying on social media allows organizations to connect with customers [4,5,6,7,8] because it enhances information sharing [9] and determines critical changes in organizational communication [5,10].

Social media is based on interactivity and content co-creation, which allows organizations to develop and maintain long-lasting online relationships with different stakeholders [6,11]. These might trigger positive influences on the online corporate reputation, general performance of employees, existing company capabilities, and organizational performance [11]. Therefore, companies effectively rely on SNS as a marketing tool to attract consumers and create positive brand associations [12]. SNS becomes a turntable for both companies and customers, communication between them being two-way oriented (i.e., switching roles as sender and receiver) to inform about products, brands, and/or co-creation processes [9,10,13,14], to facilitate touching, engaging [13], to improve customer loyalty, and to maximize the online word-of-mouth [5]. In the last five years, marketers relied even more on social media to respond to consumer complaints, foster direct customer interaction by sharing interesting stories about brands, and to promote products [5,15]. To obtain positive results, marketers are in the position of investigating and understanding customers’ experiences via SNS, especially regarding their information consumption about companies.

Customers heavily rely on SNS to obtain relevant information. For instance, in USA 53% of the population gets their news from various SNS such as Facebook (36%), YouTube (23%), Twitter (15%)., etc. [16]. The information posted on SNS in the form of news and/or press releases might be sometimes dubious and deliberately misleading. This type of content is considered to be fake news [17]. People can find themselves in the situation of creating and sharing via SNS (fake) information (or news) to the general audience [18], causing uncertainty about the true content, suspicion about accurate explanations, and eventual confidence on falsehoods [19] about companies. Customers’ cognitions, attitudes, and behaviours can be changed because of exposure to fake news [20,21].

Although literature revealed some antecedents of trust in social media information about companies, more research is needed, especially in the context of fake news proliferation. Considering the relevance of this phenomenon, literature merely highlights how fake news is studied from the consumer perspectives [22], and much research is still needed to shed light on the outcomes. There is a lack of theoretical frameworks on understanding the consequences of fake news sharing behavior [23], especially related to trust in online information about companies. Furthermore, the literature also lacks a proper understanding regarding the relationship between users’ perceived control, concentration, time distortion while browsing SNS (i.e., customers’ experiences via SNS), social media usage, sharing fake news about companies, and trust in online information.

In the last 10 years, researchers have proposed numerous theories to explain the different antecedents and outcomes of social media users’ behavior (e.g., selective exposure theory, uses and gratifications theory, social comparison theory, rational choice theory, and self-determination theory), but these efforts have only partially shed light on the complexity of this phenomenon. The paper closes this research gap by proposing a conceptual model based on a set of social media usage prerequisites (i.e., including users’ perceived control, concentration, and time distortion), social media usage, sharing fake news about companies, and trust in online information. Therefore, the research question of this study is: *Are users’ perceived control, concentration, time distortion while browsing social media networks, and sharing fake news about companies in SNS, antecedents of trust in online information about companies?*

The research is novel in that all these associations have not been investigated previously, and no other study of fake news has employed structural equation modelling (SEM) to assess the effect on trust in social media information. From a theoretical perspective, the paper enhanced the flow theory from positive psychology [24,25] by assessing the role of users’ perceived control, concentration, and time distortion (i.e., flow characteristics) in increasing social media usage, sharing fake news, and their impact on trust in social media information.

The remaining of the manuscript is structured as follows: Section 1 provides the hypothesis and conceptual model development based on a critical literature review. Section 2 includes the methodology considered for implementing the research, and Section 3 assess the results and discussions. The paper ends with conclusions, containing theoretical and managerial contributions.

## 2. Literature Review: Hypothesis and Conceptual Model Development

As highlighted over four decades ago, flow represents the “holistic sensation” encountered by individuals when they engage totally in a situation [24,25]. According to the flow theory [24], individuals experiencing flow engage in tasks while concentrating on what they are doing at a certain moment, perceiving a certain degree of control, and losing track of time regularly [24,25,26]. Research indicates that social media users are more inclined to experience flow while browsing online via SNS. Such an optimal experience can influence social media users to share different kind of news and information about organizations, brands and/or products, even fake news [10].

Perceived control constitutes the individuals’ beliefs that he/she can influence and affect the events of his/her life [27,28] to achieve a particular intended result. In psychology, a person’s perceived control (PC) refers to how much they feel in control of their environment, including the people, things, feelings, and activities around them [29]. Therefore, in a broader sense, perceived control is the “believed” control people report having during an activity. Perceived control is mainly related to individuals’ perception regarding the ease and/or the difficulty that allows them to perform and/or experience a certain behaviour relevant for them [30,31]. Within the Internet setting, perceived control refers to how web users view their capacity to effectively explore the Internet and how they engage with the web, depending on users’ inputs [32,33,34,35]. Modern technologies allow a fast and efficient response to users’ requests and complaints, thus also offering personalized feedback [36,37]. Social media provides users with a sense of perceived control by providing responsive interactive components such as chatting, replying, poking, liking, and sharing [35,38], which they are familiar with [39], and by allowing them to sign in and out at any time. These characteristics of social media networks facilitate perceived control occurrence and facilitate social media usage. Perceived control relates also to an individual’s perception of how easy it is to engage in a specific behavior [40], such as social media usage [41,42]. As a result, we posit the following:

**Hypothesis** **1** **(H_1_).** *The perceived control of social media users has a positive influence on social media usage.*

Concentration, described as the extent to which an individual is focused on a certain situation or context and/or how he/she pays attention to an ongoing activity, is a critical component of the social media user experience [38,43] and a predictor of social media usage. When individuals begin a browsing session, they are highly concentrated on the present moment and/or task they intend to accomplish, not considering information that might disturb them [24,25]. Given the variety and relevance of the information offered to users based on their interests, SNS constitutes an ideal possibility of focusing on relevant tasks. The screen limits the user’s exposure to stimuli and facilitates concentration by directing all attention to the relevant stimuli. Additionally, social media’s interactive and participatory nature stimulates users’ focus and provides immediate feedback, making it an excellent environment for users to have the best experience possible [44]. As a result of individuals paying attention to browsing activity and achieving an optimal experience, social media usage increases [38,45], so therefore we postulate that:

**Hypothesis** **2** **(H_2_).** *The concentration of social media users exerts a positive influence on social media usage.*

Users frequently lose track of time while browsing social media [38] since they are having an optimal experience [10,20,25]. Social media users spend a significant amount of time on SNS because of their immersive and gratifying nature [38], which determines stickiness [46,47] and leads to increased social media usage. Literature [48] pinpoints the link between time distortion and social media addiction, which requires a great amount of time and/or effort; it can also interfere with various daily activities or situations [49]. Regardless of whether social media users have an optimal experience or an addictive behavior while browsing, time distortion is often considered a possible prerequisite for different behaviours [26], including social media usage. Therefore, we hypothesize that:

**Hypothesis** **3** **(H_3_).** *The time distortion experienced by social media users has a positive influence on social media usage.*

Social media might be conceptualized as the collection of web-related apps developed based on the technological advancements of Web 2.0 that eases user-generated production and/or exchange of information [50]. People have always been social beings and will continue to be social regardless of the type of media they use [51]. However, without a doubt, social media platforms and apps such as Facebook, Twitter, and Instagram have affected how people interact with one another [52], but also how they engage with companies. Social media platforms contain a variety of websites (i.e., networks, wikis, video sharing sites, bookmarking sites, virtual worlds, and rating sites) that enable users to engage with peers and share information about organizations, products, and/or brands [53]. Relying on social media can generate two types of interactions: active and passive ones [50,54]. While active social media usage relates more to online behaviour enabling direct users’ interactions (i.e., like, commenting, sending messages, and interacting with other users in different ways) [55], passive usage involves observing peers without directly engaging them [56]. According to the flow theory [24,25], people who are experiencing flow (or having an optimal experience) when engaging in an activity, such as using social media, may not always be aware of the time passing which, in turn, may result in increased social media usage [38]. Social media platforms thus constitute a significant source of information for individuals and organizations [38] since it eases the fast and effortless exchange of information. Information shared over social media might be regarded as cutting a whole into ‘thin slices’ and disseminating it around [57], whereby customers can hardly assess the authenticity and credibility of the information [58].

Given the ease with which users may share and re-share data, including information, sometimes official information sources might be considered less trustworthy via social media [59]. Therefore, they are perfect for creating, consuming, and exchanging various information, including fake news [60]. When relying on social media, users also generate information within the SNS and/or the online community with whom they interact [61], so they can also share involuntarily fake news about companies. The growing usage of social media also relates to the availability and efficacy of online instruments and/or features (e.g., post, share, reshare button) that allows interaction with peers and information exchange [61]. Social media usage has been found as a predictor of spreading fake news about environmentally friendly brands on social media platforms, since it increases exposure to inaccurate information [10]. A recent study [10] examined the underlying mechanisms between social media usage and sharing fake news on SNS, including the motivations of users and their exposure to inaccurate information, and confirmed the relationship between these variables. Therefore, we postulate:

**Hypothesis** **4** **(H_4_).** *Social media usage positively influences sharing fake news about companies while browsing SNS.*

The recent fake news spread via social media can be partially explained by aspects such as non-existence of barriers/control mechanism regarding disseminated content, format of the spread information, polarization of users within so called echo chambers, the prevalence of users’ emotions, liking, and trusting only certain, well established social media sites as the most credible sources of information, etc. [57,59,62,63,64].

A search of the relevant literature regarding fake news conceptualization reveals a broad range of definitions proposed by scholars and practitioners, but also a lack of consensus. The definitional problem of fake news can be attributed to several factors [22]: (1) the phenomenon’s boundaries are blurred (i.e., the difference between fake news and other forms of misleading content, such as misinformation, disinformation, propaganda, satire, hoax, or conspiracy theories is ambiguous); (2) the fake news term is used interchangeably to describe different types of news (i.e., news produced for financial benefit or to defame others, news with a factual base but distorted to fit a specific context, and news that people simply do not feel comfortable with or disagree with); and (3) fake news was considered as both a type of misinformation (i.e., false information created unintentionally) and a type of disinformation, two distinct concepts, (i.e., false information created with the intent to deceive).

Most of the fake news definitions vary from narrow conceptualizations, according to which fake news is considered to be inaccurate, false, or grossly distorted information presented as news to deceive the audience [47,65], to broader conceptualizations, according to which fake news is disinformation that includes all forms of false, inaccurate, or misleading information designed, presented, and promoted to intentionally cause public harm or for-profit (e.g., commercial click-bait) [50,66]. Simply stated, fake news represents false, imprecise, or deceptive material portrayed as news, intentionally created to be confused with actual news, and disseminated online to mislead the audience to impact their cognition, attitude, and behaviour towards a certain event, person, or company [20]. Literature [17] also defines fake news as all types of false stories or news that are primarily published and spread on the web with the goal of deliberately misleading, deceiving, or seducing readers for financial, political, or other advantages. In line with the recommendation from the fake news literature [66], in this study, we assume the narrow definition of fake news because is considered to be more appropriate for empirical studies conducted in the consumer behaviour field [66].

The diverse varieties of fake news stories (e.g., parody, misleading content, imposter content, fabricated content, false connection, false context, manipulated content) can be classified according to different criteria, including (1) the degree of factuality, (2) the quality of the information, (3) the intention to inform, and (4) the degree of premeditated wickedness [67,68,69]. Malicious sources routinely mix and match a wide range of content formats and to generate more effective hybrid forms of fake news.

Previously limited research focused on users’ motives in sharing fake news and indicates at least two types of social media users: malign and benign ones. In the first case (i.e., malign sources), SNS users share fake news about companies being motivated by political, ideological, or financial gain and know that the information is a hoax. In the second case (benign sources), SNS users are unable to recognize the veracity of the shared information and share the bogus content without knowing it is fake. The three main motivations for benign sources to spread false information that is mistakenly believed to be true information are: self-enhancement, to be regarded as an authority or competent by other users [70]; social motivation, to interact with their community and feel a sense of belonging [71]; and altruistic motivations, to show concern for others [72] and to strive to support others [73].

Fake news can influence to a certain extent consumers’ perceptions about different companies, brands, or products [74] because it spreads farther and faster than real news [75,76,77]. For instance, the release of the Pfizer-BioNTech generated a large amount of fake news, conspiracy theories, and disinformation as it was supposed that mRNA vaccines might alter human DNA. This fake information reached in very short time millions of users of SNS such as Twitter, Reddit, and 4chan [78,79]. When an organization becomes a target of fake news, it should plan its reaction strategy meticulously to minimize the negative impact [80]. Once exposed to fake news, consumers are more likely to trust the information if it is sponsored by a famous company [74]. The mixed effects of fake news on different stakeholders and especially on consumers varies according to source intention: they might be positive if the fake news portrays the brand positively, neutrally, or negatively if the brand is the target of the fake news [81]. As trust in the news media and social media declines and concerns of disinformation and echo chambers rise, individuals must develop methods for accessing and evaluating accurate and trustworthy information not only from politics [82,83,84] but also from business.

Literature concluded that individuals’ media trust and capacity to judge between real and fake news are affected by the exposure to hoaxes in mainstream media [79,85,86,87,88]. This may result in uncertainty about past knowledge, doubts about its accuracy, and reliance on false facts [19]. From a wider perspective, the fight against the spread of fake news has become an era-defining issue [89], and the failure of this battle could lead to irreversible loss of trust in different institutions (e.g., media, government, education) [77,90,91], NGOs, and even in companies. As a result, individuals’ subsequent behaviours and judgments may be based on inaccurate information. Therefore, it is reasonable to propose the following hypothesis:

**Hypothesis** **5** **(H_5_).** *Sharing fake news about companies while browsing on social networking sites has a positive influence on trust in online information about companies.*

**Figure 1:**
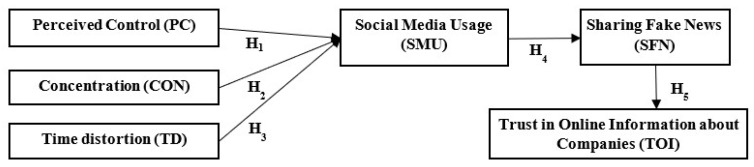
Conceptual model. Source: own development.

The conceptual model depicted in Figure 1 illustrates the relationship between users’ perceived control, concentration, and time distortion while browsing SNS, social media usage, sharing fake news about companies, and trust in social media information.

## 3. Research Methodology

### 3.1. Research Design

The research scope of the current endeavour was to assess the antecedents of trust in social media information about companies by considering users’ perceived control, concentration, time distortion while browsing SNS, and sharing fake news via social media regarding different information about companies. The data was gathered in November 2021 using an online questionnaire survey of social media users. The study has been implemented as a quantitative survey which was transposed by means of online questionnaires and was posted on various social media sites (e.g., Instagram, Facebook, and TikTok), but also to hundreds of contacts of the authors with the request that each potential respondent sends the questionnaire forward to his/her acquaintances, so that a snowball effect can be achieved. As the Official Statistics from Romania do not have data on the distribution of social media users according to gender, age, etc., a convenience sampling has been followed. Data was collected from both rural and urban social media users if they got an invitation and they agreed to answer the online questionnaire. The scope was too rich for as many potential social media users as possible, as they might have been confronted with the share of fake news about recent events. An initial pilot study on 50 respondents has been conducted, allowing a pretest of the questionnaire, to eliminate potential redundancies, and to assure a better comprehension of each statement.

From more than 1000 answers received, 986 fulfilled the first selection process (only responses from persons who indicated that they use social media daily, and that acknowledged that they encountered at least one misinformation/potential fake news spread through social media in the last 12 months were retained). In the second selection process, questionnaires with missing data, as well as those where respondents had not indicated their socio-demographic characteristics, were dismissed, thus only 922 answers remained.

The research was implemented in Romania, as the impact of fake news has strongly spread in this country especially during the COVID-19 pandemic, leading many Romanians to be hesitant regarding the vaccination [8,79,92], to fully reject it, or even deny the exitance of the COVID-19 pandemic [93]. Fake news regarding this situation has mostly spread out through social media [50], and Romanians have been encouraged to protest and/or withstand the safety measures taken by national public authorities [93] by certain narratives during the pandemic [94]. Therefore, investigating the spread of fake news through social media represents a relevant research context, thus helping organizations better understand how the effects of this tremendous phenomenon might be diminished.

In our sample, females account for around 55.7% of social media users, while males account for 44.25% (see Table 1). Most social media users are educated: 6.0% have completed high school, 7.6% have completed ten classes, 6.3% have completed vocational school, 40.6% have completed high school, 28.4% have completed university, and 11.2% have completed postdoctoral studies. Furthermore, 45.8% of respondents are younger than 30, 44.1% had were between 30 and 50, and 10.1% were over 50. Further, 43.5% of respondents registered at the time of the research had a comparably low income, 47.6% stated that they had a moderate income, and 8.9% stated that they had a high income (see Table 1).

The questionnaire was operationalized following the recommendations of Robinson [95], with scales modified from significant scientific papers in the field (see Table 2). Each item was evaluated using a five-point Likert scale (total disagreement → total agreement). The constructs (Perceived Control, Concentration, Time Distortion, Social Media Usage, Sharing Fake News, Trust in Online Information about Companies) were reflective in nature (indicators of the latent variable were correlated) and consisted of between one and five components [96]. Respondents were familiar with the key issues (measures) included in our research instrument as all of them are heavy social media users. The research was based on a self-reported online questionnaire.

### 3.2. Measurement Models Evaluation

For testing the conceptual model, we employed structural equation modelling with SmartPLS 3.0. [101] (Figure 1), all measures of the conceptual model displaying a reflective nature. Additionally, by using PLS-SEM we investigated the relations between latent variables (i.e., items) [102,103]. Different tests have been performed to assess, for instance, constructs validity and internal consistency, item loadings, average variance extracted (AVE), reliability, and discriminant validity (Table 2). It has been found that loadings exceeded the recommended minimum thresholds of 0.70, so the measured items had a proper convergence validity [102], with values ranging between a minimum of 0.747 and a maximum of 0.950. Reliability was tested with Cronbach’s α (>0.7) for acceptable confirmatory purposes [104,105].

All constructs fulfilled this threshold, so the model was found to be internally consistent. The AVE values exceed 0.5, which indicates an adequate model [106], thus supporting constructs’ convergent validity. The composite reliability values exceeded 0.7, so they are reliable [102]. Discriminant validity was assessed for each construct with the help of the Fornell-Larcker and Heterotrait-Monotrait criterion, for testing the conceptual similarity of constructs (Table 3). Based on both tools, the AVE exceeds the correlation coefficient between the component and the considered variables. As the values of the construct are below the recommended threshold of 0.9 [107], discriminant validity is given (Table 3).

Further, the collinearity level of the items in the measurement model was analysed. All VIF values are under the recommended threshold value of 5 [96]. The highest value is 3.796411 (SFN3), so there is no multicollinearity. In the following step, we applied the bootstrap procedure for testing the hypotheses and the influences between the constructs (latent variables). The hypotheses could be accepted with a significant, positive influence based on t-statistics.

### 3.3. Evaluation of the Structural Model

**Figure 2:**
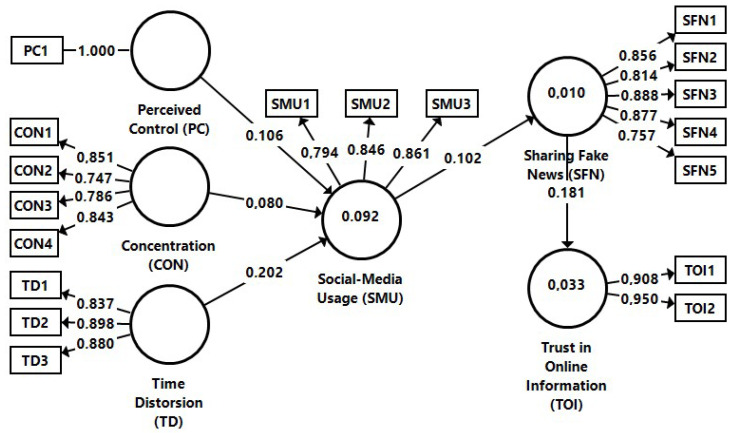
Structural model: Prerequisites of Trust in Online Information about Companies. Source: own development in SmartPLS based on the collected data.

The constructs collinearity had also to be computed to assess the structural model. The highest VIF value of the inner model was to be found of 1.566 (CON→SMU), which is under the threshold value, so multicollinearity does not represent an issue. For the saturated model, the goodness of fit is acceptable, the square root mean residual (SRMR) having a value of 0.05 < 0.08 [107]. As highlighted in Figure 2, Sharing Fake News explains 3.3% of the variance in Trust in Online Information about Companies (R^2^ = 0.033), whereas Social Media Usage only accounts for 1% of the variance in Sharing Fake News (R^2^ = 0.01). The Perceived Control, Concentration, and Time Distortion explain 9.2% of the variance in Trust in Online Information about Companies (R^2^ = 0.092), defining a moderate predicting power of the structural model [107].

## 4. Results

H_1_ assumed that the perceived control of social media users has a positive influence on social media usage. The results (β = 0.106; T-value = 2.933; *p* < 0.05) confirm the positive and strong influence of perceived control over social media usage, thus H_1_ can be accepted. H_2_ presumed that the concentration of social media users has a positive influence on social media usage. The results (β = 0.080; T-value = 2.430; *p* < 0.05) highlight that concentration has a low, but significant impact on social media usage. Therefore, we can support H_2_ (see Table 4). H_3_ inferred that time distortion experienced by social media users has a positive influence on social media usage. The results (β = 0.202; T-value = 5.986; *p* < 0.001) show the strong and positive relation between timed distortion and social media usage, so H_3_ is to be accepted. H_4_ posit that social media usage has a positive influence on sharing fake news about companies while browsing SNS. The results (β = 0.102; T-value = 2.942; *p* < 0.05) pinpoint the positive and strong influence between the considered constructs, allowing us to accept H_4_. H_5_ hypothesised that sharing fake news about companies while browsing SNS has a positive influence on trust in online information about companies. The results (β = 0.181; T-value = 5.043; *p* < 0.001) confirm the strong and positive relation between the constructs, so therefore H_5_ can also be supported (see Table 4).

## 5. Discussions

According to a Gallup survey conducted between January and June 2021 among 21,000 persons aged 15 to 24 and 40 and older from 21 nations across Africa, Asia, Europe, and North and South America, users rely on social media but do not trust it, most likely because of earlier exposure to fake news. The result of the study indicates that although individuals use social media platforms as a source of information, only 17% declare that they have a lot of trust in the accuracy of social media information [108]. These findings are explicable considering the current climate of misinformation and disinformation on social media platforms, which makes it increasingly difficult for people of all ages to discern between fact and fiction [108,109]. Numerous previous studies have been conducted on social media sharing [10,110,111] in the context of proliferation of news on social media [112]. Users share information that they perceive valuable or personal [113]. Additionally, the social media users experience (i.e., flow) can partially contribute to understanding why individuals spread fake news about companies on SNS [10]. This behaviour can therefore be considered to have important implications for trust in social media information.

When consumers come across news on social media, their level of trust is affected less by who created the item than by who shares it [114]. The experimental study demonstrates that readers’ trust in social media information is more dependent on the sharer than on who creates the post—or even on whether the content is created by a real or fictitious news agency [114,115]. Additionally, the source of the news influences whether individuals tend to share such information with their peers [110,114]. According to the findings of our study, SNS users’ trust in information about companies is affected by the dissemination of fake news, which is consistent with the findings of other studies that illustrate the detrimental impact of false news items on trust in the media companies [116], but also in and other democratic institutions [117].

## 6. Conclusions

From a theoretical perspective, our research contributes to the flow theory by offering valuable insights into the outcomes of users’ optimal experience, social media usage, sharing fake news about companies, and trust in online information about companies. Additionally, the study’s findings have significant theoretical input to the literature on fake news by indicating that social media users’ optimal experience is a predictor of sharing behaviour and is consistent with recent literature. Furthermore, our research is among the few ones combining social media usage and flow theory, thus extending even better recent research aiming at understanding how consumers react when confronted with fake news and the effects of sharing the bogus content.

From a managerial perspective, both companies and authorities must consider the impact and relevance of fake news shared on social media. They must also by proper communication foster consumers’ opinions about companies and brands, but also offer relevant information so that individuals can find the source of the information and avoid social media fake news spread. By proper communication about the facts and the true situation of a company and/or brand, the incidence of fake news might be drastically reduced. As fake news is known to dramatically affect businesses worldwide, proper understanding of its relevance and consequences, but also establishing an early warning mechanism, has the chance of allowing organizations to be even more effective in combating its effects and ex-ante informing consumers about what is real and not and how to recognize fake news. Contemporary society is confronted regularly with fake news, often consumers tending to spread them massively without proper checking their origin. Therefore, from a societal/policy makers’ perspective, authorities should take any efforts not only in combatting them, but also in flagging websites and/or social media accounts that have the potential of manipulating and misinforming citizens.

Among the limitations of the paper, we can highlight the fact that the study has been conducted only on one emerging market, namely Romania, further studies need to also consider a comparative perspective either between more countries from Central and Eastern Europe or from developed versus emerging countries. Another limitation lies in the fact that the influence of the COVID-19 pandemic, as well as the recent Ukrainian Crises, have not yet been considered, these two very recent regional events generating lots of fake news.

Future research could expand on the implications of sharing fake news about companies in different sectors, such as food, Do-It-Yourself, electronics, fashion, and shoes, etc. As fake news shared via social media has a tremendous impact on organizations and/or brands, but also on national economies, policymakers should think about developing proper legislation and best practices to help in combating such a phenomenon. Furthermore, proper and accurate information must be disseminated via national television and/or official government channels to help citizens comprehend this negative phenomenon and limit its influence on society. Future studies could also rely on comparing consumers’ perspectives about fake news and social media usage, to highlight if, for instance older consumer generations such Xers and Baby Boomers, are more inclined to spread fake news without proper checking them towards Zers or Millennials or not. An original investigation could pinpoint if customer experience and education have or do not have a relevant role in spreading fake news in emerging versus developed markets. Fake news remains a burden on contemporary society, significantly influencing citizens, countries, and organizations.

## Figures and Tables

**Table 1:** Socio-Demographic Characteristics.

Variable	Frequency	Percentage
Gender		
Female	524	55.75%
Male	408	44.25%
Education		
Primary school	8	0.9%
Gymnasium	47	5.1%
10 classes	70	7.6%
Vocational school	58	6.3%
High school	374	40.6%
College	52	5.6%
University	210	22.8%
Postdoctoral studies	103	11.2%
Age		
<30 years	445	45.8%
30–50 years	430	44.1%
>50 years	70	10.1%
Income		
Low	401	43.5%
Middle	439	47.6%
High	82	8.9%

Source: own development based on the collected data.

**Table 2:** Constructs and Items.

Construct	Item	Measure	Loading	Cronbach’s Alpha/ AVE/CR	Source
Perceived Control (PC)	PC1	While using social media, the website allows me to control the computer interaction.	1.000	1.000/1.000/1.000	Adapted from [97]
Concentration (CON)	CON1	While using social media, I am deeply engrossed.	0.851	0.833/0.652/0.882	Adapted from [38]
	CON2	While using social media, I am absorbed intensely in activity.	0.747		
	CON3	While using social media, my attention is focused on activity.	0.786		
	CON4	While using social media, I concentrate fully on activity.	0.843		
Time Distortion (TD)	TD1	When I am using social media, time seems to pass quite fast.	0.837	0.842/0.864/0.927	Adapted from [98]
	TD2	Time flies when I am using social media.	0.898		
	TD3	I frequently spend more time than anticipated on social media.	0.880		
Social Media Usage (SMU)	SMU1	On average, I spend a lot of time browsing on Instagram.	0.794	0.784/0.696/0.873	Adapted from [99]
	SMU2	On average, I spend a lot of time browsing on Facebook.	0.846		
	SMU3	On average, I spend a lot of time browsing on TikTok.	0.861		
Sharing Fake News (SFN)	SFN1	The news I shared on SNS about companies seemed accurate at the time, but later I found out it was fabricated.	0.856	0.895/0.705/0.923	Adapted from [99]
	SFN2	I did not realize that the company-related news I posted on SNS was exaggerated at the time I posted it.	0.814		
	SFN3	Initially, the company-related news I shared on SNS appeared genuine, but it was later revealed to be a hoax.	0.888		
	SFN4	The satirical news I shared on SNS about companies was presented as real news.	0.877		
	SFN5	I have shared fake news about companies on SNS having this knowledge when sharing.	0.757		
Trust in Online Information about Companies (TOI)	TOI1	I trust the information about companies that is shared online.	0.908	0.846/0.864/0.927	Adapted from [100]
	TOI 2	I trust the news about companies that is shared online.	0.950		

Note: Factor loading > 0.7; Cronbach’s Alpha > 0.7; Average variance extracted (AVE) > 0.5; Composite reliability > 0.7. Source: own development based on the literature.

**Table 3:** Discriminant validity analyses.

Fornell-Larcker Criterion						Construct	Heterotrait-Monotrait Criterion					
CON	PC	SFN	SMU	TD	TOI		CON	PC	SFN	SMU	TD	TOI
0.808						CON						
0.342	1.000					PC	0.342					
0.123	0.111	0.840				SFN	0.128	0.117				
0.229	0.179	0.102	0.834			SMU	0.253	0.204	0.117			
0.559	0.224	0.193	0.271	0.872		TD	0.647	0.242	0.223	0.325		
0.281	0.205	0.181	0.053	0.168	0.929	TSMI	0.327	0.226	0.201	0.073	0.197	

Note: CON: Concentration; PC: Perceived Control; SFN: Sharing Fake News; SMU: Social Media Usage; TD: Time Distortion; TSMI: Trust in Online Information about Companies. Source: own development based on the collected data.

**Table 4:** Path effects computed in SmartPLS.

Path Effects	Path Coefficients	Standard Deviation	T-Value	CI ^1^	*p*-Value	Hypotheses
PC→SMU	0.106	0.036	2.933	0.040–0.178	0.004 **	H_1_-Supported
CON→SMU	0.080	0.033	2.430	0.012–0.138	0.015 **	H_2_-Supported
TD→SMU	0.202	0.034	5.986	0.133–0.265	0.000 ***	H_3_-Supported
SMU→SFN	0.102	0.035	2.942	0.023–0.161	0.003 **	H_4_-Supported
SFN→TOI	0.181	0.036	5.043	0.113–0.245	0.000 ***	H_5_-Supported

Note: ** *p* < 0.01; *** *p* < 0.001; CON: Concentration; PC: Perceived Control; SFN: Sharing Fake News; SMU: Social Media Usage; TD: Time Distortion; TOI: Trust in Online Information; ^1^ CI = Confidence Interval (5–95%). Source: own development based on the collected data.

## Data Availability

Not applicable.
